# Metabonomic analysis of tumor microenvironments: a mini-review

**DOI:** 10.3389/fonc.2023.1164266

**Published:** 2023-04-14

**Authors:** Zeng Zeng, Cong-Xian Chen

**Affiliations:** ^1^ Cancer Center, Department of Ultrasound Medicine, Zhejiang Provincial People’s Hospital, Affiliated People’s Hospital, Hangzhou Medical College, Hangzhou, Zhejiang, China; ^2^ Zhejiang University School of Medicine, Hangzhou, China

**Keywords:** metabonomic, metabolomic, metabolism, tumor, drug resistance

## Abstract

Metabolomic analysis is a vital part of studying cancer progression. Metabonomic crosstalk, such as nutrient availability, physicochemical transformation, and intercellular interactions can affect tumor metabolism. Many original studies have demonstrated that metabolomics is important in some aspects of tumor metabolism. In this mini-review, we summarize the definition of metabolomics and how it can help change a tumor microenvironment, especially in pathways of three metabonomic tumors. Just as non-invasive biofluids have been identified as early biomarkers of tumor development, metabolomics can also predict differences in tumor drug response, drug resistance, and efficacy. Therefore, metabolomics is important for tumor metabolism and how it can affect oncology drugs in cancer therapy.

## 1 Introduction

Metabolism is dysregulated in tumor cells, thereby supporting the need for uncontrolled proliferation (1–3). Altered metabolism can result in different metabolic processes that can be targeted by drugs (4). Altered metabolism can also lead to unique metabolic phenotypes; thus, it can be used for diagnosing tumors earlier, selecting strategies for clinical trials, and as a biomarker of response after treatment. Many studies have demonstrated that metabolism is a vital link between environmental factors, host genes, diseases, and small metabolic molecules in tumor cells. Tumor cells reprogram metabolism and the microenvironment to support their proliferative ability (5, 6). Metabolic reprogramming of the tumor microenvironment is one of the characteristics of tumors and direction of tumor research (7). High-throughput bioinformatics technology and sequencing enable metabonomic analysis to determine the pathogenesis and etiology of tumors (8). Metabolomics is the last node of molecular pathways and last aim of omics (9).

Metabolomics includes systematic measurement of many metabolites, such as drugs, nutrients, signaling mediators, and some molecules in the urine and blood. It is also a useful tool for identifying biomarkers and drivers of tumorigenesis (10). Metabonomic analysis can measure the dynamic parameters of metabolic responses to many genetic changes and stimuli in large quantities (11). The techniques of metabolomics workflow include liquid chromatography-mass spectrometry, nuclear magnetic resonance, and gas chromatography-mass spectrometry (12, 13).

**Figure 1:**
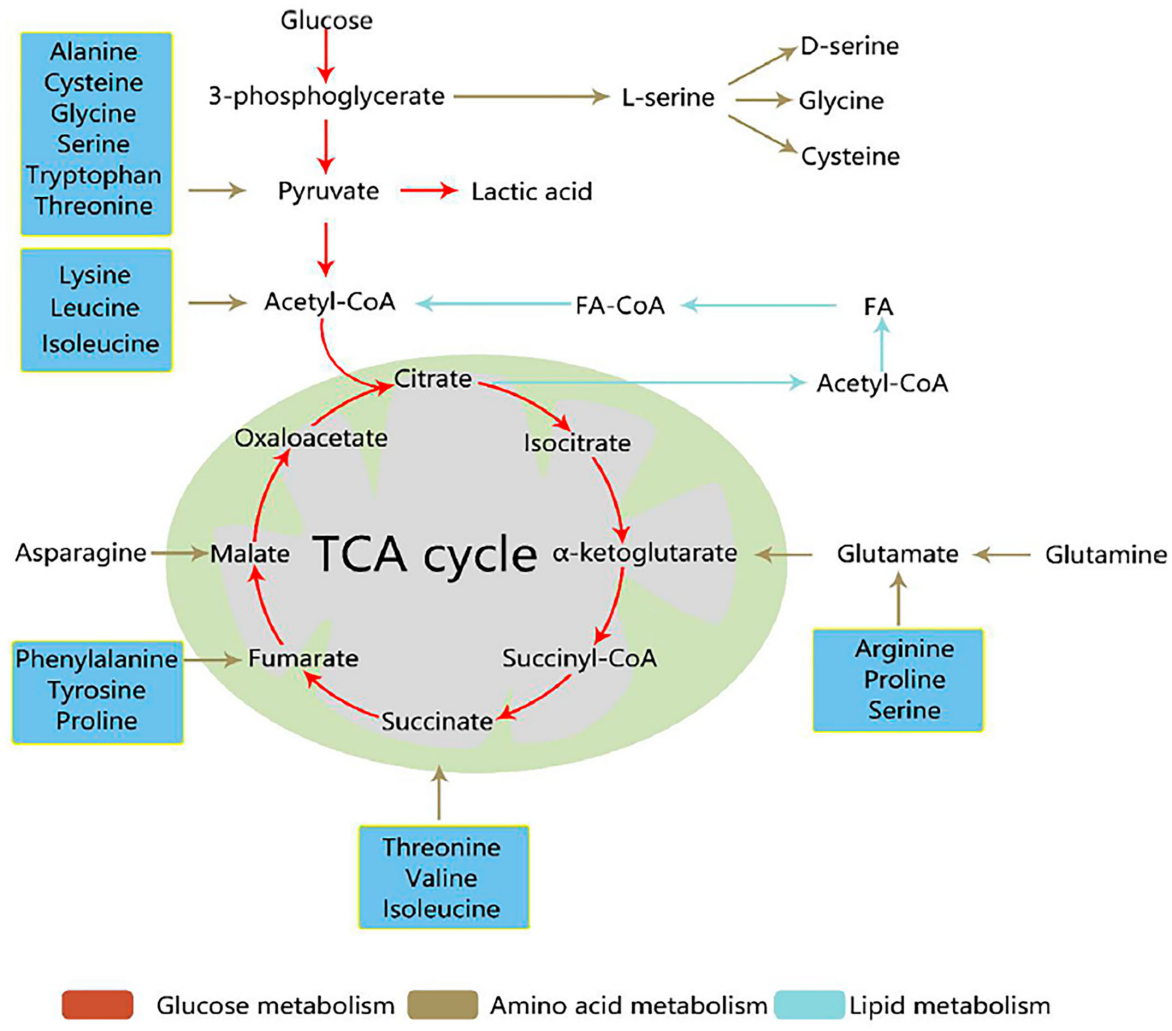
Three core metabolic pathways of cancer cells (9). Reprinted (adapted) with permission from Han, Li, Chen and Yang. (2021). Front Mol Biosci. 2021,8 (11),763902.

During tumor development, the famous part is the Warburg effect, which indicates that aerobic glycolysis pathway has a close relationship with cancer occurrence. Deregulated fatty acids and amino acids, such as serine, glutamine, and glycine can regulate metabolism to support tumor cells. Tumor cell development is closely related to three metabolic pathways (**Figure 1**) (9). Metabolomic analysis can supply tumor metabolism using the metabolic profiles of various tumor cells. This mini-review aims to explain how metabolomics can help change the tumor microenvironment, especially the three metabonomic pathways of tumors.

## 2 Definition of metabolomics

**Figure 2:**
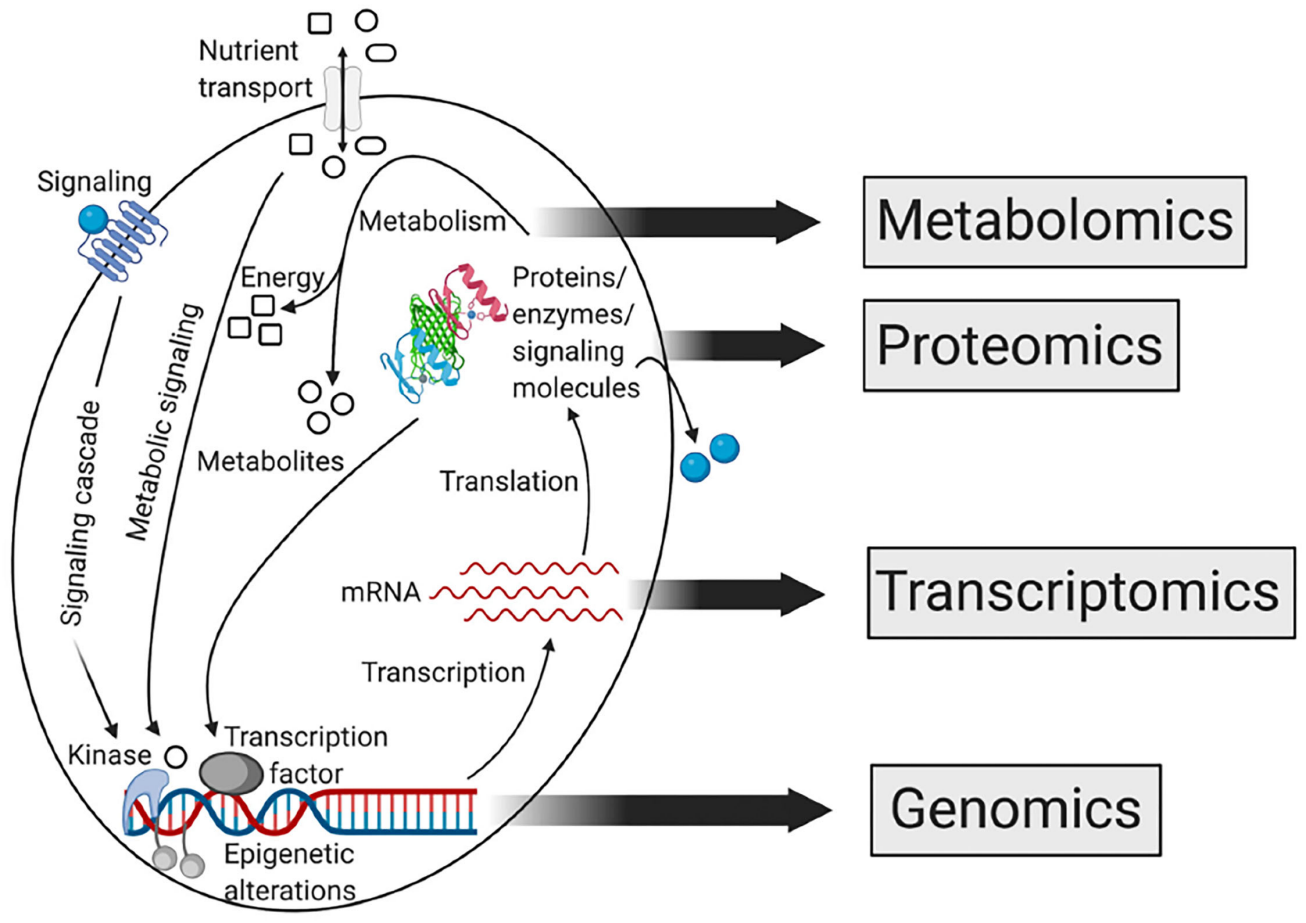
Relationship between different omics approaches of systems biology (15). Reprinted (adapted) with permission from the authors. CA: A Cancer Journal for Clinicians published by Wiley Periodicals LLC on behalf of American Cancer Society. (2021). CA Cancer J Clin. 2021,71(4),333-358.

Biological systems have many aspects, such as genomics, proteomics, transcriptomics, and metabolomics. Metabolomics is different from other omics because it can provide a functional readout of metabolic processes, and is a target phenotype assessment (14, 15) (**Figure 2**). Metabolomics can provide a readout of alterations at RNA transcription, DNA replication, and protein levels. It is a sensitive method that identifies pathologic variants under small changes in protein and significant changes in metabolite levels (16). Metabolites can alter the activity of proteins and affect almost all biological processes.

Metabolomics seems to be the analysis of some small-molecule metabolites in a biological specimen (14). It is different from standard clinical metabolites, such as urea or glucose, which rely on enzymatic reactions and a separate test for every metabolite. Recent technological advances have significantly improved metabolomics. Metabolomics can be used to produce smaller footprints.

Metabolomics involves various methods with unique advantages and disadvantages. The selection point of the appropriate method is whether a(n) targeted/untargeted method is desired. Targeted metabolomics is always used to test hypotheses and implement pictures. Untargeted metabolomics is used for biomarker discovery and hypothesis generation (17). Metabolomics deals with a complex set of molecules and is chemically diverse, while genomics, proteomics, and transcriptomics aim to identify macromolecular structures that are the sequences of amino acids, nucleotides, and so on. These chemical constituents are always limited in diversity and are well defined. Metabonomic analysis is used for data acquisition and sample preparation because sugars, lipids, organic acids, and polar molecules have various physical features. Untargeted experiments require validation with chemical standards using targeted methods. Analyzing different groups using untargeted metabolomics requires significant effort. It is important for untargeted metabolomics to facilitate biological understanding.

## 3 Metabolomic analysis of tumor metabolic pathways

### 3.1 Glucose metabolism

Owing to the huge need for tumor cell proliferation, rapid glycolysis of tumor cells is called the Warburg effect. The Warburg effect always leads to hypoxia in the tumor microenvironment (18). Glycolysis is not as effective as aerobic respiration in terms of energy supply; however, it is faster and can provide essential amino acids and intermediate metabolites of pentose phosphate in proliferating tumor cells (19). Tumor cells can be weakened or cease to use the mitochondrial aerobic oxidation pathway (20). Tumor cells are more likely to use the glycolysis pathway to produce lactic acid. The key point of the Warburg phenotype is aerobic glycolysis, which supports tumor cell proliferation by active metabolism. Glycolysis and other pathways that require glucose are also involved in glucose metabolism (21). Perroud et al. found that in renal tumor cells, enzymes that include hexokinase-1 pyruvate kinase and lactate dehydrogenase A in glycolysis can be significantly increased (22). They also found that in renal tumor cells, metabolic reprogramming is important for metabolomics, transcriptomics, and proteomics (23). Dai et al. studied breast tumors and cultured MCF-7 and T47D cells with different glucose concentrations. They demonstrated that with lower glucose levels, the proliferation of breast tumor cells can be inhibited (24). Ding et al. found that in lung tumors, glucose metabolism disorders were associated with carcinogenesis of lung tumor, thereby indicating that glucose metabolism can be a therapeutic method for lung tumor cells (25). Increased expression of metabolic enzymes can cause the Warburg effect, which is related to glycolysis. However, regulation of rate-limiting enzymes has attracted much attention from researchers in recent years (26). Researchers confirmed that the *KRAS* gene’s effect on cancer cells metabolism can occur in the method of transcriptional regulation of glycolysis enzymes and glucose transporters (26). Upregulation of glycolysis can provide potential targets in cancer therapy in the tumor cell microenvironment and many tumor-promoting signaling pathways (27).

### 3.2 Amino acid metabolism

Amino acids are required by tumor cells to increase their proliferation. They can be used not only in protein synthesis but also as metabolites that regulate tumor cell growth (28). In terms of potential biomarkers and tumor pathogenesis, multiple amino acids have been confirmed to be useful (29). Among the amino acids, serine, glycine, and glutamine have attracted attention (30). Chen et al. found that when the amino acid metabolic spectrum changes, it is always associated with gastric tumor occurrence (31). Glutamine is the most abundant free amino acid and can be found in macromolecular synthesis, energy generation, and signal transmission in tumor cells when provided with carbon atoms and nitrogen (32, 33). Glutamine can synthesize amino acids and join the tricarboxylic acid cycle. In many tumor cells, mitochondrial-dependent bioenergy production and cell biosynthesis increases the need for glutamine (34). Wettersten et al. also found higher levels of glutamine in renal clear cell carcinoma than in normal renal cells (35, 36). Additionally, arginine and aspartic acid can also be involved in reprogramming amino acid metabolism in tumors. Aspartic acid concentration is significantly negatively correlated with breast cancer when compared to gas chromatography technology and combined liquid chromatography (37). Antimetabolites that interrupt amino acid synthesis can be developed and subjected to clinical trials (38).

### 3.3 Lipid metabolism

Significant features of lipid metabolism in cancer cells include upregulated mitochondrial fatty acid β-oxidation levels and increased adipogenesis rate (39). Many cancer cells exhibit similar trends, and some metabolites involved in lipid metabolism exhibit changes. Peng et al. indicated that in the tumor microenvironments, lipid synthesis in cancer cells increased (40). Lipids provide energy to maintain membrane synthesis in tumor cells. With increased saturated fat content, the risk of some cancers increases. Dietary saturated fat can contribute to cancer progression in primary prostate cancer (41). Poczobutt et al. found that lipid metabolomic techniques can provide important information on lipid changes in many tumor cells (42). Lipid metabolism resembles a network of pathways with feedback loops and crosstalk to meet the metabolic needs of tumor cells. It can affect not only the recombination of molecules in tumor cells but also regulate crosstalk and supply high metabolic needs for the tumor cells. For example, the lipid-activated transcription factor, liver X receptor, plays a vital role in modulating the tumor microenvironment. Traversari et al. found that apoptotic cancer cells, which contain oxysterols, activate the liver X receptor in macrophages, can recruit neutrophils, and suppress dendritic cell migration; thus, resulting in immunosuppression and tolerance (43). Brandi et al. studied metabolomic detection, lipid uptake of new fat to form lipid droplets, and changes in lipid desaturation and fatty oxidation, which are related to the regulation of cancer stem cells (44). Wang et al. indicated that lipid metabolism can meet not only the standard of energy needs and biomass production of cancer stem cells, but can also activate some carcinogenic signaling pathways, such as the Wnt/β-catenin and Hippo/YAP signaling pathways (45). In summary, the lipid metabolism of tumor cells and their important roles in cancer progression and metastasis have attracted the attention of researchers.

## 4 Metabolomics in oncology drugs for cancer therapy

There are many different metabolomic markers in cancer progression, for example plasma (blood biomarkers), urine (urine biomarkers), saliva (salivary biomarkers) and cerebrospinal fluid (cerebrospinal fluid biomarkers), these markers had great importance for early diagnosing malignant tumors. Metabolomics can be used to detect tumor cell metabolites in clinical practice. It can also act as a reflection of antitumor drugs. Metabolomics can be used to improve drug efficacy and reduce adverse reactions. Zhang et al. pointed out that lipid metabolism in imidazole ketone erastin has distinct features that can induce ferroptosis and slow tumor cell growth (46). Metabolic levels can reflect gene and protein changes in terminal metabolites, once drugs begin to act on the body. Early efficacy of drugs can be evaluated using metabolomics, which enables adjustment of medication regimens.

The metabolic patterns of cancer cells change after the development of drug resistance. The same drug can induce different metabolic changes in sensitive and drug-resistant cells (47). Thus, metabolomics can be used to detect metabolic changes in cells and their response to drugs to determine whether tumor cells are resistant to drugs, and to monitor drug resistance at an early stage. Metabolomics is an easy and effective method that uses dynamic and multivariable methods to evaluate metabolic results, and to predict and assess drug resistance and sensitivity to chemotherapy. Poschner et al. used liquid chromatography mass spectrometry to characterize the levels of active estrogen, steroids, and sulfated glucose in platinum-resistant ovarian tumor cells. They demonstrated that these metabolites are highly expressed in carboplatin-sensitive cells (48). Thus, metabolomics can be used to distinguish between metabolite levels and platinum resistance. Jiye et al. found that metabolic pattern analysis can monitor drug resistance in the early stages, and can help doctors carry out follow-up treatment by observing metabolic differences between drug-sensitive and drug-resistant patients (49). Metabolomics has made significant progress in the study of drug resistance genes in tumor cells.

## 5 Conclusion

Metabolomics is of great importance in various aspects of tumor metabolism, especially in helping to change the tumor microenvironment in the three metabonomic pathways of tumors: glucose, amino acid, and lipid metabolisms. Therefore, metabolomic analysis can be used in various aspects of tumor research, such as detecting tumor cell metabolites, evaluating the effects of anti-tumor drugs, and therapeutic response.

## Author contributions

Conception of the study ZZ and C-CX, developing the search strategy ZZ and C-CX, conducting the literature search and summary ZZ, drafting the article ZZ, editing the article C-CX, and funding acquisition ZZ. All authors contributed to the article and approved the submitted version.
